# Application of ultrasound-guided intranodal lymphangiography and embolisation in cancer patients with postoperative lymphatic leakage

**DOI:** 10.1186/s12957-021-02144-2

**Published:** 2021-01-30

**Authors:** Xingwei Sun, Feng Zhou, Xuming Bai, Qiang Yuan, Mingqing Zhang, Liang Ma, Yong Jin

**Affiliations:** 1 Department of Intervention, The Second Affiliated Hospital of Soochow University, No.1055 Sanxiang Road, Suzhou, Jiangsu 215004 People’s Republic of China; 2 Department of Ultrasound Medicine, The Affiliated Suzhou Science & Technology Town Hospital of Nanjing Medical University, Suzhou, 215000 People’s Republic of China; 3 Department of Oncology, The Fourth Affiliated Hospital of Nantong University, First People’s Hospital of Yancheng, Yancheng, 224001 Jiangsu People’s Republic of China

**Keywords:** Lymphatic leakage, Lymphangiography, Ultrasound-guided

## Abstract

**Background:**

Traumatic lymphatic leakage is a rare but potentially life-threatening complication. The purpose of this study was to introduce ultrasound-guided intranodal lymphangiography and embolisation techniques for postoperative lymphatic leakage in patients with cancer.

**Methods:**

From January 2018 through June 2020, seven cancer patients (three males, four females, aged 59–75 years [mean 67.57 ± 6.11 years]) developed lymphatic leakage after abdominal or pelvic surgery, with drainage volumes ranging from 550 to 1200 mL per day. The procedure and follow-up of ultrasound-guided intranodal lymphangiography and embolisation were recorded. This study retrospectively analysed the technical success rate, operative time, length of hospital stay, clinical efficacy, and complications.

**Results:**

The operation was technically successful in all patients. Angiography revealed leakage, and embolisation was performed in all seven patients (7/7, 100%). The operative time of angiography and embolisation was 41 to 68 min, with an average time of 53.29 ± 10.27 min. The mean length of stay was 3.51 ± 1.13 days. Lymph node embolisation was clinically successful in five patients (5/7, 71.43%), who had a significant reduction in or disappearance of chylous ascites. The other two patients received surgical treatment 2 weeks later due to poor results after embolisation. All patients were followed for 2 weeks. No serious complications or only minor complications were found in all the patients.

**Conclusions:**

Ultrasound-guided intranodal lymphangiography and embolisation were well tolerated by the patients, who experienced a low incidence of complications. Early intervention is recommended for cancer patients with postoperative lymphatic leakage.

## Background

Traumatic lymphatic leakage is a rare but potentially life-threatening complication for cancer patients [1, 2]. Abdominal and pelvic surgery is the main cause of lymphatic leakage [3–5]. Lymphatic vessel ligation is the standard treatment for high-output lymphatic leakage; however, cancer patients often find this procedure difficult to tolerate [6–8].

Intranodal lymphangiography and lymphatic leakage embolisation is a new technique that has emerged in recent years [9, 10]. Here, we introduced ultrasound-guided intranodal lymphangiography and embolisation techniques to address postoperative lymphatic leakage. Seven cancer patients with lymphatic leakage after abdominal or pelvic surgery were treated with this new technique, and the procedure and follow-up results are reported.

## Materials and methods

This study was approved by the ethics committee of our hospital. Informed consent was obtained from the patients.

Seven patients received interventional therapy for lymphatic leakage in our department from January 2018 to June 2020. We retrospectively analysed their clinical data (Table 1).

**Table 1:** Procedural data of all study patients

Patient no.	Age (years)/sex	Surgery	Procedure times (min)	Preoperative drainage (mL)/day	Postoperative drainage (mL)/day	Complications	Treatment measures
1	59/M	Subtotal gastrectomy	63	750	700		Surgical treatment
2	73/M	Radical prostatectomy	68	870	50		
3	67/F	Cystadenoma resection	51	1200	1100		Surgical treatment
4	61/F	Hysterectomy	48	550	None		
5	72/M	Radical gastrectomy	59	650	30	Infection	Anti-infective therapy
6	66/M	Kidney transplantation	43	1100	None	Chronic diarrhoea	Symptomatic treatment
7	75/F	Hysterectomy	41	600	20		

**Fig. 1:**
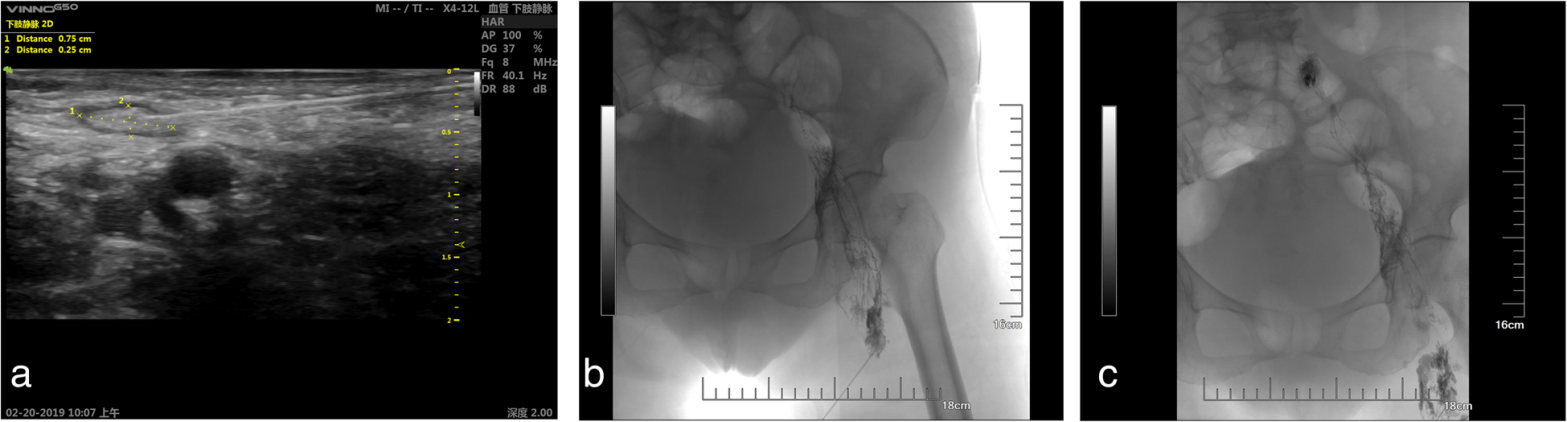
**a** Ultrasound-guided inguinal lymph node puncture. A 22-G puncture needle (white arrow) was guided by ultrasound to puncture the inguinal lymph nodes (black star). The circular hypoechoic area on the lower right is the femoral artery (white star). **b** Intranodal lymphangiography. The 22-G puncture needle (white arrow) punctured the inguinal lymph node (black star). The black arrows show the lymphatic vessel. **c** Lymphatic leakage embolisation. The white arrow shows the location of the lymphatic leakage. The black arrows indicate the path of the lymphatic vessel

In the supine position, appropriate inguinal lymph nodes were identified using ultrasound and prior CT or MRI images. A high-frequency probe (> 7.5 MHz) was used to detect superficial lymph nodes. A 60-mm, 25-G Cathelin needle (Terumo Europe, Leuven, Belgium) was used to puncture the inguinal lymph nodes under ultrasound guidance (Fig. 1a). The ultrasound-guided needle tip was positioned into the inguinal lymph nodes at the junction of the hilum and the cortex to allow sufficient contrast agent uptake and minimise extravasation [11], and iodised oil (Lipiodol; Guerbet, Roissy, France) was gently injected manually at a rate of approximately 1 to 2 mL every 5 min under intermittent fluoroscopy (dedicated lymphangiogram pump or, more commonly, an advanced anaesthesia injection pump with a rate of 0.2–0.4 mL/min). A peripheral iv catheter was used to connect the syringe to the Cathelin needle, and the assistant kept the needle as still as possible. The lymph nodes showed a small dark area that drained into the lymph vessels through its continuous branches (Fig. 1b). The total dose of iodised oil should be limited to 0.25 mL/kg. The X-ray showed that the iodised oil accumulated in the abdomen. A large amount of iodized oil leaked from the lymphatic vessels into the abdominal cavity, consistent with the drainage site from which the lymphatic fluid leaked (Fig. 1c).

Approximately 2 mL 5% glucose water was used to push the remaining iodine oil to the accumulation site. *N*-butyl cyanoacrylate (NBCA) (B. Braun Melsungen AG, Melsungen, Germany) and iodised oil were mixed well (1:3–1:4), providing a long enough injection time; the mixture was injected manually at a rate of approximately 1 to 2 mL every 5 min (alternatively “dedicated” 0.2 to 0.4 mL/min) under intermittent fluoroscopy by the puncture needle at the aggregation site for embolisation of the lymphatic leakage, and 2 mL 5% glucose water was used again to push the mixture out of the inguinal lymph nodes. The needles were then removed, and the puncture site was sterile bandaged.

Ultrasound or CT was used for postoperative follow-up.

## Results

Ultrasound-guided inguinal lymph node puncture and lymph node angiography were technically successful (Table 1). Angiography revealed that all seven patients had leakage, and all patients underwent embolisation. Clinical efficacy was defined as a significant reduction in daily leakage (more than 80%) over 2 weeks. In five patients (5/7, 71.43%), embolisation was clinically successful. Chylous ascites improved significantly in three patients and disappeared in two patients. The other 2 patients received surgical treatment afterwards. The patients were hospitalised for 3 to 7 days, with an average of 5.2 days. One patient developed chronic diarrhoea after the operation, and one patient developed puncture site infection; these conditions improved after symptomatic treatment. In order to assess daily leakage, drains were left in place for at least 14 days postinterventionally. No case of lymphoedema of the lower extremities occurred after the intervention, and no serious complications were found in these patients.

## Discussion

Lymphatic leakage seriously affects the prognosis of patients and delays further treatment [12–16]. Janco et al. described that surgical treatment is helpful in avoiding metabolic complications, and ligation or suture of the leakage site is recommended [17]. However, surgical treatment for lymphatic leakage is a more aggressive approach, and it is often difficult for patients with cancer to tolerate another operation after abdominal or pelvic surgery [18, 19].

The technique of lymphatic embolisation was originally proposed by Cope et al. [20]. The relevant literature notes that the effective rates of lymphangiography and embolisation for postoperative lymphatic leakage are 56% and 86%, respectively; thus, the procedures can be used as alternatives to surgical treatment [21–23].

**Fig. 2:**
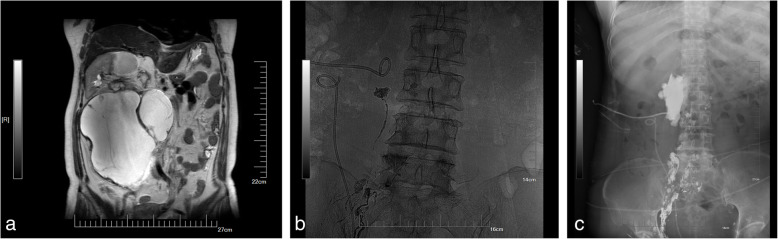
Intranodal lymphangiography and embolisation were performed to treat lymphatic leakage after abdominal surgery (patient 3). **a** Preoperative MRI shows a large cystadenoma with a maximum diameter of 16 to 18 cm. **b** The white arrow is a drainage tube placed at the time of surgery. The white star shows the leakage of iodized oil and aggregation on the right side of the spine. **c** The mixture was slowly injected at the site (black star) under fluoroscopy

In this study, the clinical effective rate was 71.43% (5/7), which was consistent with the reports of Matsumoto et al. [21–23]. In our study, drainage volume decreased significantly in three patients, and drainage gradually disappeared in two patients. We saw poor results in the remaining two patients. One of these patients was diagnosed with a giant abdominal cystadenoma and underwent surgical resection (Fig. 2a). Abdominal distension occurred after the operation in this patient. Combined with the MRI examination results, lymphatic leakage was considered; hence, abdominal puncture drainage was performed, and the daily drainage volume was as high as approximately 1200 mL. Because the patient had high-flow leakage and had previously undergone surgery, another operation was risky. Ultrasound-guided intranodal lymphangiography and embolisation were performed after interdisciplinary consultation. During the operation, iodised oil leakage accumulated on the right side of the abdomen (Fig. 2b, c). After the contrast medium was flushed out of the iodised oil, a 1:3 iodised oil NBCA mixture was used for lymphatic leakage embolisation. There was persistent lymphatic leakage after the operation, with a daily drainage volume of 700 mL, which was lower than before. The patient underwent further surgical treatment 1 week later.

For patients with poor clinical effects, preoperative lymphangiography can better determine the leakage site, provide an effective reference for surgical treatment, and facilitate the localization and ligation of the target lymphatic vessels during the operation [23].

It has been reported that the NBCA iodised oil diluent was mixed evenly at a ratio of 1:2 [24]. In our study, NBCA iodised oil diluent was used and mixed evenly at a ratio of 1:3 to 1:4. We believe that this concentration can provide a long injection time, allowing sufficient advancement of the NBCA glue in the lymphatic networks. In addition, 5% glucose water was used to push the residual iodised oil to the aggregation site before embolisation to prevent the mixture from accumulating rapidly in the lymph nodes.

Kim et al. retrospectively evaluated the complications of 24 patients who underwent successful lymphatic embolisation. Kim et al. noted that in view of the well-known serious mortality and incidence of untreated chylothorax, lymphatic embolisation may be a feasible option for the treatment of chylothorax [9].

In our study, ultrasound-guided puncture of inguinal lymph nodes was used. Good ultrasound guidance techniques are key to the success of the operation. The incidence of complications in the study was 28.57% (2/7); all complications were mild and controllable, including one case of chronic diarrhoea and one case of puncture site infection; all complications improved after treatment. Another case of diarrhoea in our study was considered to be caused by other factors. After a follow-up of 2 weeks, no serious complications were found in the patients.

In patients with pulmonary insufficiency (PaO_2_ lower than 60 mmHg) or with a right-to-left cardiac shunt, iodised oil embolisation risks aggravating a pulmonary embolism or causing a cerebral embolism. Although the incidence of such complications is low, they are fatal. Such patients were outside the scope of this study [25].

The sample size of this study was limited, because postoperative lymphatic leakage is a rare condition. However, this case series clearly demonstrates the feasibility of ultrasound-guided lymph node angiography and lymphatic embolization as a therapeutic option especially for elderly cancer patients who cannot easily tolerate surgery again. The efficacy and safety of this technique needs to be validated in larger prospective trials.

## Conclusion

Ultrasound-guided intranodal lymphangiography and embolisation can be used as a supplement to surgical treatment. The procedure offers the advantages such as minimal trauma, high tolerance by patients, and a low incidence of complications. Therefore, this early intervention is recommended for cancer patients with postoperative lymphatic leakage.

## Data Availability

Research data can be obtained from the corresponding author upon reasonable request.
